# A Clinical Phrase Book; in English and German, Containing the Usual Questions and Answers Employed in Examining and Prescribing for Patients; Questions in Asking for and Buying Medicines, Etc.

**Published:** 1853-07

**Authors:** 


ART. VI.—*A Clinical Phrase Book;* in English and German, containing the usual questions and answers employed in examining and prescribing for patients; questions in asking for and buying medicines, etc. With an English-German and German-English Pronouncing Lexicon, of all the words occurring in the Phrases, with the chief technical terms of medical writers and apothecaries; grammatical appendix, table of idioms, &c., Designed to aid physicians and surgeons in Hospitals, Alms House and private practice; also druggists and pharmaceutists, in dispensing their prescriptions. By MONTGOMERY JOHNS, M. D. Philadelphia: Lindsay & Blackiston. 1853.

We regard this as a most useful and valuable book for medical men. The increasing emigration of Germans to the western portions of the Union, makes such a work as the Clinical phrase book almost indispensable Of this portion of the book the author says: "The questions are arranged in the order commonly followed in what is technically called *taking the case;* my model being that modification of Ricamier's plan given in Stilla's Elements of General Pathology, and being chiefly questions committed to paper by the bed-side from time to time as propounded. The answers, it is hoped, will be found to present that latitude which is generally noticed in similar replies; and both will suffice to elicit that sort of information relative to *subjective* symptoms necessary to correct diagnosis. * * * * The grammatical and lexicographical portions of the book were added to render the work complete in itself—for those who wish to study the language; the beautiful copper-plate, of the German current hand, will be found useful both to the physician and apothecary as well as to all who wish to acquire the useful and elegant accomplishment of writing the German lang:age in its own character. Of the utility of books of phrases, as aids in acquiring a knowledge of a foreign tongue, and especially for accelerating the progress of those whose aim is an early habit of speaking it fluently, there exists but one opinion, and that too decidedly favorable to need any apolegetic comments here." * * *

"The increasing numbers of the German emigrant population, (only exceeded by the Irish) in our country generally, and in our Atlantic cities especially, where they remain for sometime after arriving among us, render their language an object of peculiar interest to all connected with the hospitals and eleemosynary institutions of the United States; the autumnal fevers, pneumonias, and catarrhs, the frequent cases of phthisis, and what may be called the febris advenarum, and typhoid fever, are all chiefly presented to the student of medicine, in the persons of German or Irish patients. To aid him and his professor, as well as the general practitioner, this little book is now offered to the profession.

"Two sections, on the examination of patients, either women or children, are added at the close of the Phrases, as they could not be incorporated into the body of the volume. It is hoped that nothing will here be found to offend the most sensitive delicacy, where the parties are necessarily woman and her physician. I have sought to bear in mind all along the caution of Cicero: 'retinenda est igitur hujus generis verecundia, præsertime natura ipsa magistra et duce.'"

"To Rab. Ab. Rice, by whom the phrases were first translated, to Mr. Julius E. Müller, of St Thomas College, Leipsic, who kindly revised the whole, to Mr.

Charles Sander of the University of Halle, who aided in drawing up the Lexicons, to my brothers, who have kindly shared with me every portion of the work, and the labor of reading the proofs, I am deeply indebted."

We cheerfully bear testimony to the fidelity of the work. We have given it a great deal of examination and study, and have found this little volume, which may be carried in the pocket, a highly useful and improving book. We have used a large portion of the general preface of the author, in our notice of the volume, because it sets forth clearly and accurately the merits of the work.

In the preface to the lexicons, the author says: "the reader will better understand the plan and scope of these two lexicons when he is informed what they do not contain. Within the brief space of 150 pages he will hardly expect to find all the words both of the English and German languages, though we promise him the *sources* of the immense vocabulary belonging to each. The dictionary in each part is PRONOUNCING, being based upon Oehlschläger's recent Pronouncing German Dictionary. From his pronounciation I have in no case departed, except where the equivalent sounds might be represented by combinations of letters more familiar to an English eye, than those chosen by the German author." After giving a list of words, whose compounds are not found in the work, the author proceeds: "Secondly, the reader *will find* all the terms denoting his profession, business; the elements of surgical, medical, obstetrical, chemical, pharmaceutical, therapeutical, pathological, physiological, and anatomical nomenclature; the various particles employed in forming questions, answers, hypothetical and declamative sentences; the words denoting the various acts of the living body in health and in disease; the words entering into colloquial conversation; the prefixes and suffixes used in forming compound words; the auxiliaries of all the verbs." This is the scope of the not unambitious attempt to include within the brief space of 150 pages the elements of the language of Aschenbrenner, Chelius, Liebig, Mülder, Mitscherlich, Zetemeyer, Vogel, Müller, Valentin, Arnold, Förg, Henlé, Huschke, Bischoff, Wagner, Nægele, Carns, Trigand, and the many equally eminent scholars whom Germany has given to medicine and its kindred sciences. If the reader desires to form some approximative notion of the resources of this language, its variety and power of endless self-multiplication, let him turn to the word Wasser (water) in Adler's large 8vo Dictionary, and examine the *seven* columns of the compounds of this one word; Tod (death) with its *three* columns, or the articles Blut, Licht, Erd, (blood, light, earth); or the compounds of haus (house), three closely printed columns, and he will see a meaning in the language of Klopstock which hecould in no other way discover:

"Are we separated and forever, consequently, unmixed, as when Tacitus, our critic, observed us in the ancient times?"

Hence the utility of a dictionary which shall present within the compass of a pocket-volume the primitive materials of this vast vocabulary of 'immer neuer und doch deutscher Wendung.' But many compounds and derivatives, likely to occur in ordinary conversation, or having a peculiar signification not deducible from a knowledge of their components, or strictly technical, have been unhesitatingly admitted in both parts of the dictionary. The several terminations and prefixes are inserted in their alphabetical places, with their significations."

"The first part, English-German, has been constructed on the same general principles. Two peculiarities, however, need remark, viz., that the lexicon contains a number of *latin* words with their German equivalents: 1, all the names of the Materia Medica included in the last edition of the Nationnl Pharmacopœia; 2nd, the most usual of the latin technical terms employed in chemical language. The English-German part is chiefly drawn from Adler's reprint of Flügel's work, published at London under his name, though compiled by Hiemann, Feiling, and Oxenford, and characterised by his American editor as "at present the most complete, and the most judiciously prepared manual of its kind in England."

As to the reliance that may be placed upon this work, the author says, and we think truly: "Much care has been bestowed upon this department to select the words most closely equivalent to the English term taken in its medical signification, to secure *exactness* and *definiteness*, where in general the most annoying looseness and unsatisfactoriness prevail."

Of the pronounciation adopted throughout the book, Oehlschlager says: "I shall only insure those persons, who make use of this Dictionary, and pronounce according to the pronounciation given, after having carefully studied the directions, that they will be understood by *all classes* of Germans, however differently they themselves may pronounce."

We have thus let the author set forth the characteristics of this Clinical Phrase Book, because we are satisfied of the correctness of his descriptions. An extended examination of the book has satisfied us, not only of its great value, but of its utility. As an aid to the medical man in his intercourse with German patients, we could scarcely say too much in praise of the work, and we warmly commend it to the attention of medical men. Through the knowledge that may be obtained from the Cinical phrase book, they may not only facilitate their practice among Germans, but may hold intercourse with the great minds of German literature.

These books may be obtained at the bookstore of E. Maxwell & Co.

